# α1-antitrypsin and its C-terminal fragment attenuate effects of degranulated neutrophil-conditioned medium on lung cancer HCC cells, *in vitro*

**DOI:** 10.1186/1475-2867-4-7

**Published:** 2004-11-21

**Authors:** Inga Zelvyte, Tim Stevens, Ulla Westin, Sabina Janciauskiene

**Affiliations:** 1 Lund University, Department of Medicine and Otholaryngology, University Hospital Malmo, 20502 Malmo, Sweden

## Abstract

**Background:**

Tumor microenvironment, which is largely affected by inflammatory cells, is a crucial participant in the neoplastic process through promotion of cell proliferation, survival and migration. We measured the effects of polymorphonuclear neutrophil (PMN) conditioned medium alone, and supplemented with serine proteinase inhibitor α-1 antitrypsin (AAT) or its C-terminal fragment (C-36 peptide), on cultured lung cancer cells.

**Methods:**

Lung cancer HCC cells were grown in a regular medium or in a PMN-conditioned medium in the presence or absence of AAT (0.5 mg/ml) or its C-36 peptide (0.06 mg/ml) for 24 h. Cell proliferation, invasiveness and release of IL-8 and VEGF were analyzed by [^3^H]-thymidine incorporation, Matrigel invasion and ELISA methods, respectively.

**Results:**

Cells exposed to PMN-conditioned medium show decreased proliferation and IL-8 release by 3.9-fold, p < 0.001 and 1.3-fold, p < 0.05, respectively, and increased invasiveness by 2-fold (p < 0.001) compared to non-treated controls. In the presence of AAT, PMN-conditioned medium loses its effects on cell proliferation, invasiveness and IL-8 release, whereas VEGF is up-regulated by 3.7-fold (p < 0.001) compared to controls. Similarly, C-36 peptide abolishes the effects of PMN-conditioned medium on cell invasiveness, but does not alter its effects on cell proliferation, IL-8 and VEGF release. Direct HCC cell exposure to AAT enhances VEGF, but inhibits IL-8 release by 1.7-fold (p < 0.001) and 1.4-fold (p < 0.01) respectively, and reduces proliferation 2.5-fold (p < 0.01). In contrast, C-36 peptide alone did not affect these parameters, but inhibited cell invasiveness by 51.4% (p < 0.001), when compared with non-treated controls.

**Conclusions:**

Our data provide evidence that neutrophil derived factors decrease lung cancer HCC cell proliferation and IL-8 release, but increase cell invasiveness. These effects were found to be modulated by exogenously present serine proteinase inhibitor, AAT, and its C-terminal fragment, which points to a complexity of the relationships between tumor cell biological activities and local microenvironment.

## Background

The relationship between inflammation, innate immunity and cancer is widely accepted. Early in the neoplastic process, inflammatory cells and their released molecular species influence the growth, migration and differentiation of all cell types in the tumor microenvironment, whereas later in the tumorigenic process neoplastic cells also divert inflammatory mechanisms, such as metalloproteinase production, and chemokine/cytokine functions to favour tumor spread and metastasis [1-3]. Human polymorphonuclear neutrophils (PMN) comprise 50–70% of circulating leukocytes and induce inflammatory reactions that can be either cytotoxic for tumour cells or promote tumour growth and metastasis [4,5]. For example, animal studies show that circulating neutrophils isolated from tumor-bearing animals reduce the number of metastatic foci in the lung. Other studies reveal that neutrophils stimulate tumour cell attachment to endothelial monolayers [6,7] and neutrophil-released cytokines, growth and angiogenic factors enhance tumour growth and spread [8]. Most of the neutrophil-induced tumour-promoting effects are attributed to their abilities to release proteases. Neutrophil degranulation results in the release of serine proteases, such as elastase, cathepsin G and protease-3, which may contribute to the activation of matrix metalloproteinases (MMPs) that mediate tumor cell invasiveness [9-12]. Recently, Schwartz and co-workers found that neutrophil-secreted factors can activate pro-MMP-2, which is important in ECM degradation and tumor cell invasion [14]. It is now also apparent that tumor cells can be sources of proteolytic enzymes themselves. For example, some cells possess immunoreactive neutrophil elastase [15], which is suggested to play a role in increasing tumor cell invasiveness of surrounding tissue [16]. Levels of immunoreactive neutrophil elastase in tumour extracts are reported to be an independent prognostic factor for patients with breast cancer and non-small cell lung cancer [17-19].

A large number of studies support the notion that proteases play an important role in the progression of malignant tumors. Therefore, the expression of proteinase inhibitors is considered to be an antimalignant event [20]. Alpha-1 antitrypsin (AAT), a major inhibitor of human serine proteases in serum, is produced mainly by the liver, but also by extrahepatic cells, including neutrophils and certain cancer cells [21,22]. AAT is an acute phase protein and its concentration rises up to 3–4-fold above normal during acute inflammation [23]. Several types of cancer, including non-small cell lung adenocarcinoma, have been associated with increased serum levels of AAT [20,24]. Clinical studies have shown that high circulating levels of AAT directly correlate with tumour progression [24-26]. Recently we also found that plasma levels of AAT are significantly elevated in lung cancer patients and, particularly in cases with metastases [27]. The role of AAT is poorly understood in tumor diseases although it is suggested that it can inhibit tumor cell growth and invasion, *in vitro *[28]. Moreover, not only the native, inhibitory form of AAT, but also conformationally modified, non-inhibitory forms are suggested to play a role in modulating tumour growth and invasiveness. For example, Kataoka and co-workers have shown that the C-terminal fragment of AAT can enhance the growth and invasiveness of human pancreas adenocarcinoma cells, *in vivo *[29]. Our in vitro studies revealed that the C-terminal fragment of AAT, corresponding to amino acid sequence 358–396 (C-36 peptide), induces breast tumour cell proliferation and invasiveness [30]. Recently we found that C-36 peptide induces neutrophil chemotaxis, adhesion, degranulation and superoxide generation, *in vitro *[31], which we propose may indirectly affect tumor cell biological activities, *in vivo*. Together these findings prompted us to design an experimental model *in vitro *that would allow us to evaluate how neutrophil-derived products themselves and in combination with exogenously added native AAT or its C-36 peptide influence lung cancer HCC cell growth, invasion and metastatic properties.

## Results

### HCC cell proliferation

**Figure 1:**
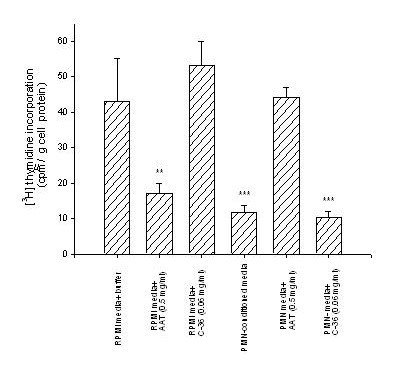
[^3^H]Thymidine incorporation assay. The HCC cells were exposed to AAT (0.5 mg/ml) or its C-36 peptide (0.06 mg/ml) in a regular medium or in a PMN-conditioned medium for 20 h following addition of [^3^H] thymidine for 4 h. Each bar represents mean ± standard deviation from three separate experiments with three repeats in each. *** p < 0.001, ** p < 0.01.

To examine the effect of the degranulated PMN-conditioned media alone or supplemented with exogenously added native AAT (0.5 mg/ml) or its C-36 peptide (0.06 mg/ml) on lung cancer HCC cell proliferation, we measured DNA biosynthesis using a [^3^H]-thymidine incorporation assay (Fig. 1). HCC cells cultured in PMN-conditioned medium for 24 h decrease proliferation by 3.9-fold, (p < 0.001), while under the same experimental conditions cells exposed to PMN-conditioned medium supplemented with native AAT show no changes in proliferation compared to control cells cultured in a regular media. HCC cells exposed directly to native AAT decrease [^3^H] thymidine incorporation by 2.5-fold, (p < 0.01) compared to control cells cultured in a regular medium. Thus, our data show that both PMN-conditioned medium and AAT loose their inhibitory effects on cancer cell proliferation when used in combination. Under the same experimental conditions, the C-36 peptide of AAT added either directly to HCC cells or as a supplement with PMN-conditioned medium did not exert any significant effects on HCC cell proliferation relative to controls (Fig. 1).

### HCC cell invasiveness

**Figure 2:**
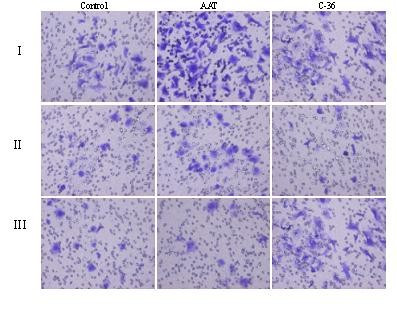
HCC cell invasiveness after exposure to PMN-conditioned media, AAT or C-36 peptide separately and in combinations. HCC cells cultured in a regular medium (I) and in a PMN-conditioned medium (II) alone or supplemented with AAT or C-36 peptide (this figure represents one of three independent experiments).

To characterize lung cancer cell properties after their exposure to PMN-conditioned medium with and without added AAT or its C-36 peptide, we performed cell invasiveness assays. As illustrated in Fig. 2 and Table 1, cells cultured in a PMN-conditioned medium show markedly increased cell invasiveness (by 56.8%, p < 0.001) compared to controls. When PMN-conditioned medium is supplemented with native AAT or its C-36 peptide, its effects on cancer cell invasiveness are diminished by 41.5%, and 77%, (p < 0.001), respectively, compared to PMN medium alone. It must be pointed out that HCC cells cultured in a regular medium in the presence of C-36 peptide decreased cell invasion by 51.4%, (p < 0.001), while native AAT showed slight, but not significant up-regulation of cell invasiveness, compared to controls. Together these data show that native AAT as well as its C-36 peptide abolish the capacity of PMN-conditioned medium to stimulate HCC cell invasiveness.

**Table 1:** Quantitative evaluation of HCC cell invasiveness (migrated cells/per view, in ten randomly selected fields).

Stimulus	HCC cells in a regular medium			HCC cells in PMN-conditioned medium		
	mean^♠)^		SEM	mean		SEM
		
Control (medium)	27.8	±	0.85	43.6***	±	1.6
AAT (0.5 mg/ml)	32.7	±	2.1	25.5***	±	1.99
C-36 (0.06 mg/ml)	13.5***	±	1.5	10.0***	±	0.86

♠ mean and standard error of 3 independent experiments; *** -p < 0.001

### Release of Vascular Endothelial Growth Factor (VEGF) and interleukin-8 (IL-8)

**Figure 3:**
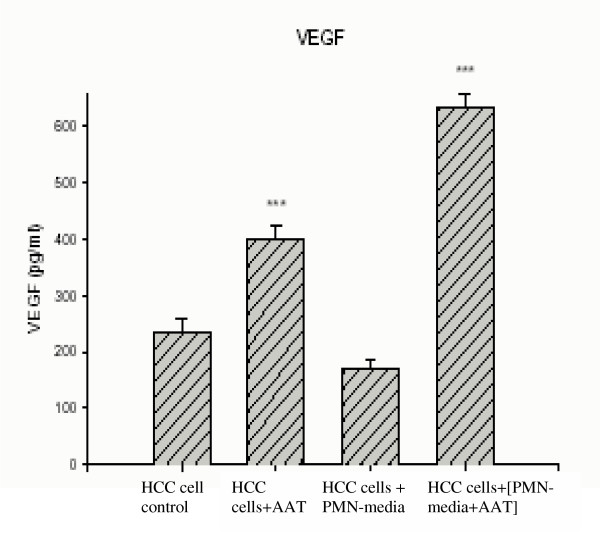
VEGF release from HCC cells alone or exposed to PMN-conditioned medium with and without addition of AAT. Each bar represents the mean ± standard deviation of six repeats from two separate experiments with three repeats in each. *** p< 0.001

**Figure 4:**
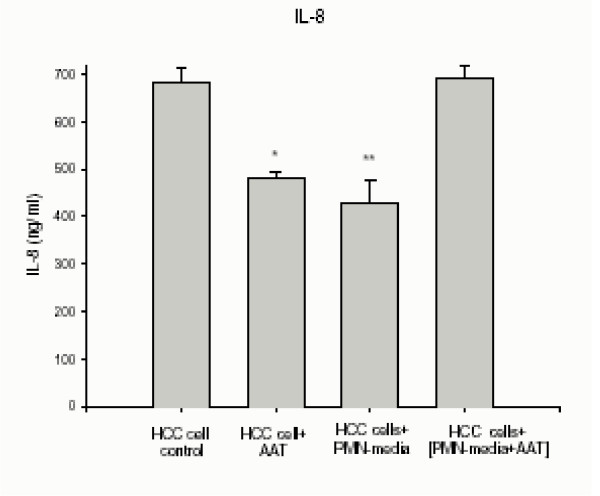
IL-8 release from HCC cells alone or exposed to PMN-conditioned medium with and without addition of AAT. Each bar represents the mean ± standard deviation of six repeats from two separate experiments with three repeats in each. ** p < 0.01, * p < 0.05.

Vascular endothelial growth factor (VEGF) and chemokine IL-8 are factors produced by tumor cells as well as by neutrophils [32-34], and are known to correlate with lung cancer angiogenesis *in vivo *[35]. We analysed for VEGF and IL-8 released by HCC cells alone or cultured in PMN-conditioned medium with and without added AAT or C-36 peptide for 24 h (Fig. 3 and 4). As shown in Fig 3, AAT increases VEGF release by 1.7-fold, p < 0.001, and inhibits IL-8 by 1.4-fold, p < 0.01, relative to control cells. Under the same experimental conditions, cancer cells cultured in a PMN-conditioned medium supplemented with AAT increase VEGF and IL-8 release by 3.7-fold, p < 0.001 and 1.6-fold, p < 0.01, respectively, compared to PMN-condition medium alone. In addition, HCC cells in a PMN-conditioned medium show a slight (~27%), but not significant, decrease in VEGF levels compared to cells grown in regular medium. It must be noted that C-36 peptide had no influence on VEGF and IL-8 levels under any experimental conditions used (data not shown).

### Enzymatic activity in PMN-conditioned medium

**Figure 5:**
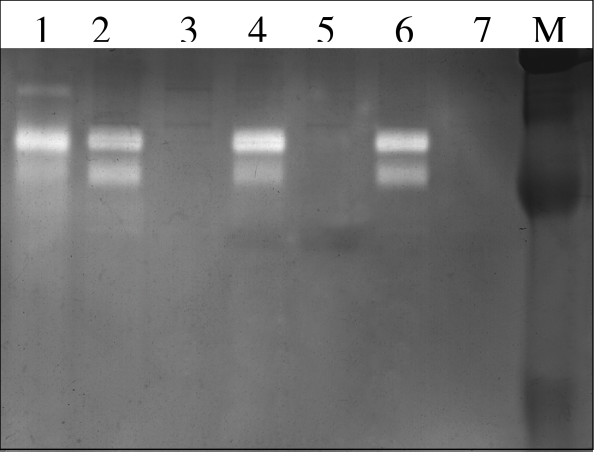
Effects of PMN-conditioned medium alone and supplemented with AAT or C-36 peptide on HCC cell gelatinolytic activity. Lane 1-PMN-conditioned medium alone; lanes 2 and 3, PMN-conditioned medium supplemented with C-36 peptide and AAT, respectively; and incubated with cancer cells for 24 h, lane 4-PMN-conditioned medium incubated with HCC cells alone; lanes 5 and 6 – HCC cells exposed to C-36 peptide and AAT, respectively, in a regular medium; 7-HCC cells alone. This figure represents 1 of 3 separate experiments performed under the same experimental conditions.

**Figure 6:**
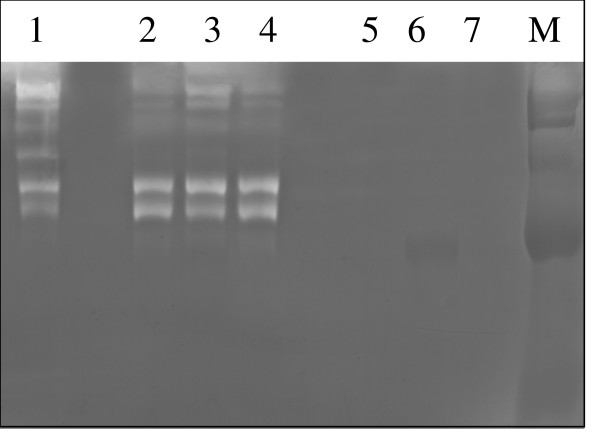
Effects of PMN-conditioned medium alone and supplemented with AAT or C-36 peptide on HCC cell caseinolytic activity. Lane 1-PMN-conditioned medium alone; lanes 2-PMN-conditioned medium supplemented with C-36 peptide and incubated with HCC cells for 24 h, line 3-HCC cells exposed to C-36; lane 4-; PMN-conditioned medium supplemented with AAT and incubated with HCC cells for 24 h, lane 5-HCC cells exposed to AAT; lane 6-PMN-conditioned medium incubated with HCC cells; lane 7-HCC cells alone. This figure represents 1 of 3 separate experiments performed under the same experimental conditions.

Neutrophil degranulation results in a large release of proteases, and to verify this we monitored proteolytic activity in PMN-conditioned medium by zymography, an electrophoretic technique used for the qualitative evaluation of proteases. As illustrated in Fig. 5 and 6 line-1, PMN-conditioned medium reveals a specific pattern of gelatinolytic and caseinolytic activity at a position between 72 and 180 kDa, which most likely represents neutrophil collagenase (MMP-8) (75 kDa), gelatinase A (MMP-2) (72 kDa), gelatinase B (MMP-9) (92 kDa) and several MT-MMPs, such as MT-MMP-2, -5 (72–75 kDa). Lung cancer cells grown in regular medium (Fig. 5 and 6, line-7) or treated with AAT and C-36 peptide show no gelatinolytic (Fig. 5, lines 6, 5) and caseinolytic activity (Fig. 6, lines-5, 3). PMN-conditioned medium incubated with HCC cells for 24 h manifested a decrease in certain enzymatic activities (Fig. 5, line 4, Fig. 6, line 6) compared to PMN media alone (Fig 5 and 6, line-1). However, no further changes in enzymatic activity profiles were observed when HCC cells were grown in PMN-conditioned medium supplemented with native AAT (Fig. 5, line 3 and 6 line-5) or C-36 peptide (Fig 5 and 6, line-2).

### Molecular profile of AAT in HCC cell culture supernatants

**Figure 7:**
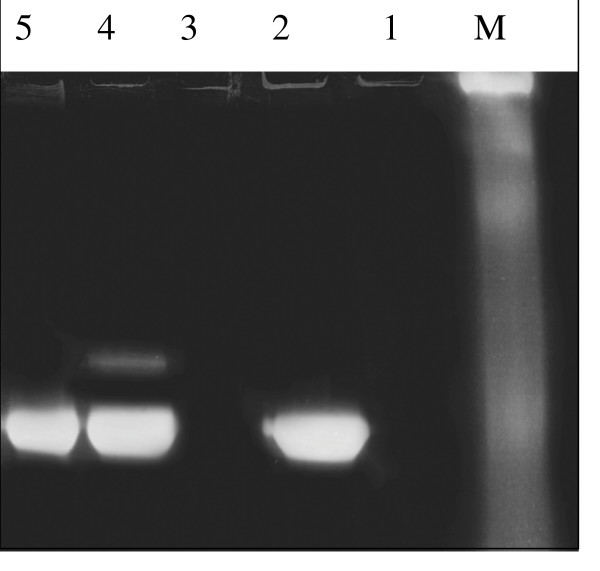
HCC cell culture medium alone and in the presence of AAT studied by Western blot analysis. Cell culture supernatants were applied to 10% SDS-PAGE and immunoblotted with polyclonal antibody against AAT. M, molecular size markers (myosin-205 000, β-galactosidase-123 000, bovine serum albumin-79 000, carbonic anhydrase-45 700), lane 1-HCC cells alone, lane 2-HCC cells stimulated with AAT, lanes 3 and 4-HCC cells cultured in PMN-conditioned medium alone and supplemented with AAT, respectively lane 5-ATT done. The arrow indicates complexed AAT. The AAT profile shown in this figure represents one of three similar experiments.

Cell culture supernatants collected from HCC cells cultured in a regular medium or in PMN-conditioned medium with and without addition of AAT were analysed for AAT by 7.5% SDS-PAGE followed by immunoblotting using polyclonal antibody against human AAT. As shown in Fig. 7, line-2, AAT added to HCC cells was unchanged in amount and form compared to native AAT alone (Fig. 7, line-5), whereas AAT added as a supplement into PMN-conditioned medium is altered in the distribution of its forms: in addition to monomeric AAT, a complexed form of AAT can be detected (Fig. 7, lane-4).

## Discussion

Local and systemic inflammatory mediators derived by tumor-associated PMN and tumor cells are suggested to play a role in the development of lung tumors [36,37]. Different studies have reported an increase of proteolytic activity in cancer and have suggested a role of proteases in tumor progression and metastasis [38-40]. For example, Guner and co-workers have shown that the plasma concentration of elastase in patients with malignant lung carcinoma is 10-fold higher compared to benign lesions of the lungs [41]. An increased concentration of neutrophil elastase is found to be closely associated with progression of non-small cell lung cancer (NSCL). In parallel, an increase in levels of proteinase inhibitors, such as AAT, has also been reported in tumor cases, including lung tumors. These elevated levels of proteinase inhibitors are attributed to the inflammatory reactions accompanying the tumorigenesis [42,43].

PMN, the most abundant circulating blood leukocytes, are potent effectors of inflammation and release a wide profile of serine and metalloproteinases [44]. Infiltration of tumors with PMN is associated with a favourable prognosis in some studies in humans, however for individual patients there is no predictable relationship between PMN infiltration and cancer prognosis [45,46]. Thus, in the present study we aimed to investigate how degranulated neutrophil-conditioned medium alone and supplemented with the serine proteinase inhibitor, AAT, or its C-terminal peptide (C-36), affects functional activities of a non-small cell lung adenocarcinoma cell line (HCC), *in vitro*.

We found that PMN-conditioned medium expresses multiple effects on lung cancer HCC cell functional activities (decreases in cell proliferation and IL-8 release and stimulation of cell invasion through the ECM membrane), but has no significant effects on VEGF release. These dual neutrophil activities toward cancer cells have been described by other investigators. It has been suggested that through the release of cytokines, chlorinated oxidants and defensins, neutrophils may cause direct tumor killing [47,48] while in parallel, through release of serine proteinases and MMPs, breakdown of basement membranes and ECM and increase in tumor invasiveness are promoted [49]. Our findings that PMN-conditioned medium decreases HCC cancer cell proliferation and IL-8 release, but increases invasiveness, in part support the hypothesis that neutrophils can both, inhibit and promote tumor cell growth and invasiveness. In parallel experiments, we found that PMN-conditioned medium supplemented with AAT loses its effects on HCC cell invasion, proliferation and IL-8 release, but enhances VEGF. In contrast, C-36 peptide added to PMN-conditioned medium abolished the effects of medium on cell invasiveness, but had no significant influence on medium effects on cell proliferation, IL-8 and VEGF release.

The major function of AAT is to inhibit neutrophil derived serine proteases, particularly neutrophil elastase [50]. A local increase of proteinases and PMN elastase-AAT complexes has been demonstrated in bronchoalveolar lavage from patients with lung cancer [51]. AAT is also known to interact with other components of degranulated-PMN. For example, AAT binds to defensins and neutralizes their effects on cell cytotoxicity and migration [52]. In our *in vitro *model, AAT added to degranulated PMN-conditioned medium occurs in a complexed form. Thus, the modulating effects of AAT on PMN-medium activities toward HCC cancer cells can be attributed to AAT alone, but also to its interaction(s) with components of the medium. Verification of the effects of AAT on separate components released by degranulated PMN needs further investigations.

It is important to point out that AAT alone as well as in combination with PMN-conditioned medium induced VEGF release from HCC cells. VEGF is one of the most potent angiogenic molecules, regulating both angiogenesis and vascular permeability, and hence promotes tumor progression and development in NSCLC [53]. Many compounds, including anti-VEGF antibody and anti-VEGF receptor antibody have been developed as VEGF inhibitors and these compounds were reported to inhibit growth of a wide variety of tumor cell lines *in vitro *and in animal models, *in vivo *[54]. Therefore, our in vitro findings that AAT expresses potent effects on VEGF release allow classify AAT as a tumor promoter. Contrary data exist for a relationship between the levels of AAT and cancer advancement. Some investigators propose that AAT plays a protective role in tumorogenesis. For example, Finlay and co-workers showed that AAT might be directly involved in breast cancer progression by acting as a tumor suppressor [55]. Yavelow and co-workers have shown that AAT inhibits human breast cancer cells MCF-7 growth [56] and AAT was also shown to block the activation of pro MMP-2 and tumor cell invasion [11]. However, immunohistochemical studies revealed that patients with AAT-positive colon, gastric and lung adenocarcinomas had a worse prognosis than AAT-negative ones [57-59]. Together these findings suggest that AAT may play multiple roles in cancerogenesis in vivo, in addition to its role as proteinase inhibitor.

During tumor progression, inflammatory cells produce large amounts of IL-8, an autocrine growth factor [53,60]. Levels of IL-8 have also been shown to correlate with lung cancer angiogenesis. Current knowledge of the degranulation process of neutrophils *in vitro *suggests that the chemokine IL-8 promotes rapid release of all neutrophil granular stores after cell exposure to cytoskeleton-disrupting agents [61]. In the present study, we found that the amount of IL-8 released by HCC can be reduced by AAT or PMN-conditioned medium alone, whereas these inhibitory effects are abolished when AAT and PMN-conditioned media are added together. Based on our findings, we hypothesize that IL-8 and other angiogenic factors released from HCC cells are regulated by PMN components, which may potentially initiate tumor cell activities or induce anti-tumor responses dependent on the tumor cell microenvironment.

AAT is the main inhibitor of neutrophil elastase and proteinase 3 [62] and it has been reported that an imbalance between AAT and neutrophil elastase may predispose to lung cancer development [63]. Patients who carry the deficiency allele of AAT have a significantly higher risk of developing squamous cell or bronchoalveolar carcinoma of the lungs [64]. On the other hand it was reported that strong expression of AAT in lung adenocarcinoma correlates with poor prognosis [65], although it was not shown whether the inhibitory activity of AAT was normal and whether elastase levels were high among this group of patients. Moreover, AAT is a good substrate for MMPs: neutrophil collagenase, gelatinase-B, stromelysin-1 and -3, and matrilysin can effectively cleave AAT [66-69]. The cleavages by these MMPs occurs at peptide bonds within AAT active site loop, resulting in a generation of cleaved forms of AAT. Comparative proteome analysis performed to identify protein alterations in plasma of prostate, lung and breast-cancer patients showed significant elevation of AAT and its N-terminal fragment [70], which points to a role for different molecular forms of AAT in cancer progression. Recent studies provide evidence that the C-terminal fragment of AAT may enhance tumor growth and invasiveness *in vitro *and *in vivo *[29,30]. We found that the C-36 peptide displays striking concentration-dependent pro-inflammatory effects on human neutrophils, including induction of neutrophil chemotaxis, adhesion, degranulation and superoxide generation [31]. Interestingly, in our HCC cancer cell model, C-36 peptide reduced HCC cell invasiveness and also abolished PMN-conditioned medium-induced cell invasiveness, whereas there were no significant effects on other parameters measured, such as cell proliferation, VEGF and IL-8 release.

## Conclusions

Our data show that AAT in various molecular forms expresses differential effects on lung tumour cell responses and provide an experimental evidence for complexity in the interactions of neutrophil-released molecular species and lung cancer cells.

## Materials and methods

### AAT and its C-terminal peptide

Native, purified human plasma AAT was purchased from the Clinical Chemistry Department (UMAS, Malmö). The quality of AAT preparations was confirmed by 7.5% SDS-PAGE and determination of anti-elastase activity as described by Gaillard et al. [71]. Synthetic C-terminal fragment of AAT (C-36, corresponding to residues 359–394) was obtained from Saveen, Biotech AB (Denmark) and was greater than 98% purity. The C-36 peptide was prepared in sterile, endotoxin-free Tris buffered saline (0.015 M Tris, pH 7.4 containing 0.15 M NaCl) just before use. The endotoxin content in AAT was tested by quantitative E-TOXATE Assay (Sigma, USA). According to international standards no more than 0.08 enzyme U/ml of endotoxin is allowed to be present in AAT solutions [72]. In our experimental model we used AAT preparation with endotoxin levels below 0.05 enzyme U/ml. If necessary, AAT samples were purified using Detoxi-Gel™ endotoxin removing gel (Pierce, Rockford, IL, USA) according to the manufacturers recommendations.

### Neutrophil isolation

Neutrophils were isolated from the peripheral blood of healthy donors using Polymorphprep™ (Axis-Shield PoC AS, Oslo, Norway) according to the manufacturers recommendations. Neutrophils were harvested as the lower cellular band above the red cell pellet. Residual erythrocytes were removed by a hypotonic lysis using ice cold 0.2% NaCl (w/v) for 30 s, followed addition of an equal volume of 1.6% NaCl to restore isotonicity. The neutrophil purity was typically 90% as determined by an AC900EO AutoCounter (Swelab Instruments, Sweden) and cell viability exceeded 95% according to trypan blue staining.

### Preparation of PMN-conditioned medium

PMN-conditioned medium was prepared by degranulating neutrophils (5 × 10^6 ^cells/ml) according to Videm and Strand [72]. Briefly, isolated neutrophils were incubated on a shaker for 1 h at 37°C and immediately after on ice for 3 min, and centrifuged at 240 × *g *for 10 min at 4°C. Supernatants were collected and analyzed for the cytokine release and enzyme activity.

### Cell culture

Human non-small cell lung carcinoma (HCC) cells were established from the lung of 54-year old women with non-small cell lung the adenocarcinoma type (DSMC No. ACC 534). The cells were routinely grown at 37°C in a humid air containing 5% CO_2_, in 125-cm^2 ^flasks in RPMI-1640 medium, supplemented with 10% FBS, 100 IU/ml penicillin, and 100 μg/ml streptomycin. Medium was changed every 2–3 days. Prior to experiments HCC cells were seeded into 12-wells plates at a density of 3 × 10^5 ^cells/ml, and cultured until confluent. After, cells were washed and a serum-free medium containing test substances was added for 24 h. Two sets of experiments were designed: the first, in which HCC cells were exposed to native AAT (0.5 mg/ml) or C-36 peptide (0.06 mg/ml) in a RPMI-1640 and the second, in which the cells were cultured in PMN-conditioned medium alone or supplemented with native AAT or C-36 peptide.

### [^3^H] Thymidine incorporation assay

HCC cells were incubated with test substances for 20 h. [^3^H] Thymidine was then added (0.2 μCi/ml) for a further 4 h at 37°C. The medium was then aspirated, the cells were washed twice with 0.5 M NaCl and incubated for 5 min with 5% trichloroacetic acid. The cells were then washed with distilled water, dissolved in 1 ml 0.5 M NaOH, neutralised with 200 μl HCl and radioactivity determined in a β-counter (Packard 300CD liquid scintillation spectrometer; Packard Instruments). Protein concentration in cell lysates was determined by the Lowry method using HSA as a standard.

### Matrigel invasion assay

Cell invasion assay was performed in an invasion chamber, a 24-well tissue culture plate with 12 inserts. The inserts contain an 8 μm pore size polycarbonate filters over which is placed a thin layer of ECMatrix™. HCC cells were suspended in a serum free medium containing 1 × 10^6 ^cells/ml alone or together with test substances. Each suspension was added to the upper chamber of an ECM Invasion system. Medium containing 10% of fetal bovine serum (FBS), serving as a chemoattractant, was added to the lower chamber. The chambers were incubated at 37°C in 5% CO_2 _for 24 h. Invasive cells were stained and counted using microscope (Olympus BX41, PC program Olympus MicroImage) at a 100 × magnification. The number of cells per field, from 10 randomly selected fields, are presented.

### Enzyme activity assay

HCC cells were cultured alone or stimulated with test substances for 24 h. Cell free supernatants were then analysed by zymography using 10% and 12% Tris-Glycine gels containing 0.1% gelatin or β-casein, respectively (NOVEX, Invitrogen Life Technologies, UK). Briefly, supernatants were diluted in a sample buffer and separated by electrophoresis at 125 V for 90 min. The gels were then renatured and developed over night in a developing buffer (NOVEX, Invitrogen Life Technologies, UK) at 37°C. After washing, the gels were stained with Coomassie Blue R-250. Proteases that can utilize casein or gelatine as a substrate showed up as clear zones in the gels.

### Endothelial growth factor (VEGF) and IL-8 analysis

HCC cell supernatants were analysed for the VEGF and IL-8 levels by using commercially available quantitative ELISA kits (R&D systems, Minneapolis, USA) according to manufacturers instructions. The lowest detectable concentration for VEGF and IL-8 was 15.6 pg/ml and 10 pg/ml, respectively.

### Immunoblotting

HCC cells supernatants were analysed by 10% SDS-PAGE gels and when transferred to a polivinylidene fluoride (PVDF) membrane (Millipore, Millipore Corporation, Bedford, MA 01730) using a semi-dry immunoblot transfer system. The blot was visualised using polyclonal rabbit antibody to human AAT (1:500) (DAKO, A/S, Denmark), secondary horseradish peroxidase-conjugated swine anti-rabbit antibody (1:800) (DAKO, A/S, Denmark) and peroxidase substrate, DAB (3,3-diaminobenzidine tetrahydrochloride) (Sigma, USA).

### Statistics

The differences in the means of experimental results were analysed for their statistical significance with the one-way ANOVA combined with a multiple-comparisons procedure (Scheffe multiple range test), with an overall significance level of α = 0.05. Statistical Package (SPSS for Windows, release 11.0) was used for the statistical calculations.

## List of abbreviations

AAT – alpha1-antitrypsin; C-36 – C-terminal fragment of AAT; FBS – fetal bovine serum; NSCLC – non small-cell lung cancer; MMPs – matrix metalloproteinases; MT-MMP – membrane type matrix metalloproteinase; PBS – phosphate buffered saline; PMN – polymorphonuclear neutrophil; VEGF – vascular endothelial growth factor.

## Authors contributions

Inga Zelvyte – AB, Tim Stevens – JY, Ulla Westin – JY, Sabina Janciauskiene ES, FG
